# Risk of Primary Liver Cancer Associated with Gallstones and Cholecystectomy: A Meta-Analysis

**DOI:** 10.1371/journal.pone.0109733

**Published:** 2014-10-07

**Authors:** Yanqiong Liu, Yu He, Taijie Li, Li Xie, Jian Wang, Xue Qin, Shan Li

**Affiliations:** Department of Clinical Laboratory, First Affiliated Hospital of Guangxi Medical University, Nanning, Guangxi Zhuang Autonomous Region, China; Sanjay Gandhi Medical Institute, India

## Abstract

**Background:**

Recent epidemiological evidence points to an association between gallstones or cholecystectomy and the incidence risk of liver cancer, but the results are inconsistent. We present a meta-analysis of observational studies to explore this association.

**Methods:**

We identified studies by a literature search of PubMed, EMBASE, Cochrane Central Register of Controlled Trials, and relevant conference proceedings up to March 2014. A random-effects model was used to generate pooled multivariable adjusted odds ratios (ORs) and 95% confidence intervals (CIs). Between-study heterogeneity was assessed using Cochran’s Q statistic and the *I^2^.*

**Results:**

Fifteen studies (five case-control and 10 cohort studies) were included in this analysis. There were 4,487,662 subjects in total, 17,945 diagnoses of liver cancer, 328,420 exposed to gallstones, and 884,507 exposed to cholecystectomy. Pooled results indicated a significant increased risk of liver cancer in patients with a history of gallstones (OR = 2.54; 95% CI, 1.71–3.79; *n* = 11 studies), as well as cholecystectomy (OR = 1.62; 95% CI, 1.29–2.02; *n* = 12 studies), but there was considerable heterogeneity among these studies. The effects estimates did not vary markedly when stratified by gender, study design, study region, and study quality. The multivariate meta-regression analysis suggested that study region and study quality appeared to explain the heterogeneity observed in the cholecystectomy analysis.

**Conclusions:**

Our results suggest that individuals with a history of gallstones and cholecystectomy may have an increased risk of liver cancer.

## Introduction

Primary liver cancer mainly includes hepatocellular carcinoma (HCC), which originates in liver cells, and intrahepatic cholangiocarcinoma (ICC), which arises from the intrahepatic bile duct [1]. The worldwide burden of primary liver cancer for 2012 was estimated at 782,000 new cancer cases [2]. It ranks as the fifth most common incident cancer in men and the ninth in women [2]. Owing to its poor prognosis, it is the second commonest cause of death from cancer worldwide [2]. The above data highlight the importance of a better understanding of risk factors related to liver cancer development. However, the etiology of this disease remains largely elusive, apart from known relationships with hepatitis B or C virus infection, alcohol, aflatoxins, liver cirrhosis, and diabetes [3], [4].

It has been hypothesized that gallstones (i.e., cholelithiasis) and cholecystectomy are associated with an increased risk of several cancers, especially the risk of rectal cancer [5], pancreatic cancer [6], and colorectal cancer [7], [8]. Gallstones are known to induce biliary inflammation, and cholecystectomy is typically followed by dilation of the common bile ducts and elevated bile duct pressure, which also results in chronic inflammation [9]. The link between chronic inflammation and cancer is well established [10]. It has also been proposed that gallstones and cholecystectomy result in the accumulation of bile and secondary bile acids, in particular, deoxycholic acid [11]–[15], and that bile acids can act as carcinogens [16].

Several epidemiological studies have investigated the association between gallstones, cholecystectomy, and liver cancer [17]–[31]. However, the existing results are controversial. Most studies have reported a positive relationship between gallstones and liver cancer [17]–[19], [21], [22], [24], [26], [27], [29], [30], but one failed to demonstrate a significant association [23]. With regard to cholecystectomy, several studies suggested a significant increased risk of liver cancer [18], [20], [24], [25], [27], [28], [31], whereas others demonstrated a nonsignificant adverse effect [17], [19], [21], [26], [29].

No meta-analysis has previously been published on the relationship between gallstones or cholecystectomy and the incidence risk of liver cancer. The aim of this detailed meta-analysis was to summarize the association between cholecystectomy, gallstones, and the risk of developing liver cancer in observational studies. A better understanding of these relationships may highlight the need to consider additional intervention methods in this area.

## Methods

This study complies with the guidelines of the PRISMA (Preferred Reporting Items for Systematic Reviews and Meta-Analyses) checklist and flow diagram [32] (Checklist S1).

### Data sources and search strategy

We searched PubMed, EMBASE, and Cochrane Central Register of Controlled Trials (CENTRAL) in the Cochrane Library for all relevant articles on the risk of liver cancer in patients with history of gallstones or cholecystectomy. The search was performed in each database from time of inception until March 12, 2014 by two independent investigators (Y.L. and S.L.). Medical subject heading terms and keywords used in the search included “cholecystectomy”, “gallbladder surgery”, “gallstones”, “gallstone”, “cholelithiasis”, “cholecystolithiasis”, “choledocholithiasis” combined with “HCC”, “hepatocellular carcinoma”, “liver cancer”, “liver tumors”, “liver neoplasms”, “hepatic carcinoma”. No language restrictions were imposed. We also reviewed the abstracts submitted to major gastroenterology and hepatology conferences (annual meeting of the *American College of Gastroenterology*, *American Association for the Study of Liver Diseases*, *Digestive Diseases Week*; *World Congress of the International Hepato-Pancreato-Biliary Association*) between 2009 and 2013. The reference lists in all identified articles were checked for further relevant articles.

### Eligibility criteria and study selection

Studies considered in this meta-analysis met all the following inclusion criteria: (1) cohort or case-control; (2) focus of the study was a history of gallstones or cholecystectomy; (3) end point was liver cancer incidence; (4) provided multivariate-adjusted relative risks, with corresponding 95% confidence intervals (CIs) for events associated with gallstones or cholecystectomy vs. controls, at least adjusted for three of eight factors (hepatitis B or C virus infection, smoking, alcohol, cirrhosis, diabetes, body mass index, age, gender). Primary exclusion criteria were cross-sectional studies, literature reviews, commentaries, editorials, and case reports. Studies were also excluded where adjusted relative risks and/or CIs had not been provided, or they did not fulfill the inclusion criteria. When there were multiple studies of the same population, only data from the most recent comprehensive report was included. Two authors (Y.L. and Y.H.) independently evaluated all records by title and abstract and subsequently retrieved and assessed, in detail, the full text of any potentially relevant articles using the above eligibility criteria. Disagreements regarding eligibility were resolved through discussion and by referencing the original report.

### Data abstraction and quality assessment

Data were independently abstracted onto a standardized form by two investigators (T.L. and L.X.). Disagreements were resolved through consensus, referring back to the original article. The following data were collected from each study: first author’s name, year of publication, country of the population studied, mean age, study duration, number of patients with gallstones or cholecystectomy studied, number of incident cases of liver cancer, adjustment factors, and multivariable adjusted relative risk estimates and their 95% CIs (relative risk [RR] for cohort studies, odds ratios [OR] for case-control studies, or standardized incidence rates [SIR] for studies comparing the rates of observed to expected cases). Because the liver cancer incidence is low (≤10%) and the estimated effects are small, odds ratios (ORs) can be considered close approximations of risk ratios [33].

The methodological quality of the case-control and cohort studies was initially assessed independently by two authors using the Newcastle-Ottawa scale (NOS) [34] (X.Q. and S.L.). Disagreements were resolved through discussion with an additional adjudicator (J.W.) who was completely blinded to the study until a consensus was reached. Observational studies were scored across three categories and allocated a maximum of 9 points: selection (up to 4 points), comparability (up to 2 points), and outcome (up to 3 points). The overall study quality was arbitrarily defined as poor (score 0–3), fair (score 4–6), or good (score 7–9).

### Outcomes assessed

The primary analysis focused on assessing the risk of liver cancer in patients with a history of gallstones, and risk of liver cancer in patients with a history of cholecystectomy. Additionally, based on information available from individual studies, we assessed sex-specific differences in risk estimates.

### Data synthesis and statistical analysis

We used the random-effects model described by DerSimonian and Laird [35] to calculate summary ORs and 95% CIs. Heterogeneity was first tested by Cochran’s Q test, and a *P*-value <0.10 was considered suggestive of significant heterogeneity [36]. To estimate the proportion of total variation across the studies that was due to study-related factors rather than chance, the *I^2^* statistic was calculated [37]. An *I^2^* less than 30% was considered as low, 30%–60% as moderate, 60%–75% as substantial, and more than 75% as considerable [38].

To explore sources of heterogeneity between the combined studies, we performed subgroup analyses based on study design (case-control vs. cohort), study location (Asian, Europe, and the U.S.), and study quality (good vs. fair, and poor). In addition, a restricted maximum likelihood-based random-effects meta-regression analysis was performed to assess heterogeneity associated with the aforementioned factors [39].

Sensitivity analyses were conducted to assess the robustness of the results by sequential omission of individual studies [40]. Publication bias was assessed graphically using a funnel plot and quantitatively using Egger’s regression asymmetry tests [41]. A 2-tailed *P*-value less than 0.05 was considered statistically significant for all analyses except for the Cochran’s Q test. All analyses were performed using STATA, version 12.0 (StataCorp, College Station, TX, USA).

## Results

### Study selection, study characteristics, and quality

**Figure 1:**
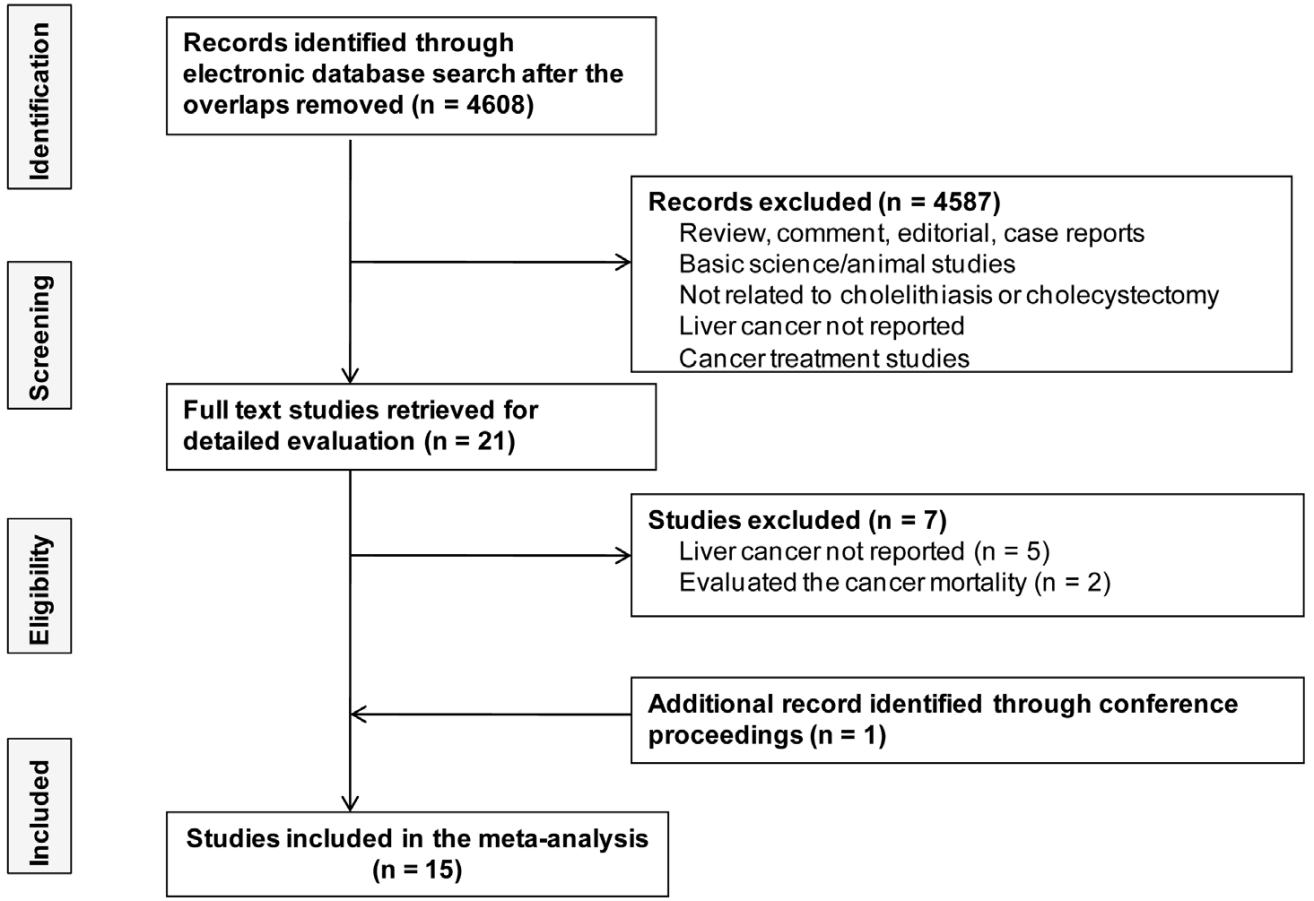
Flow chart depicts the selection of eligible studies.

Fig. 1 summarizes the process of study identification, exclusion, and inclusion. Table S1 shows the list of excluded studies from the full text studies review. At least 15 studies fulfilled all inclusion criteria and were included in the meta-analysis [17]–[31]. Fourteen articles were published in full [17], [18], [20]–[31], and one was in abstract form [19]. There were five case control studies [18], [22], [23], [26], [27] and 10 cohort studies [17], [19]–[21], [24], [25], [28]–[31]. Individual study characteristics are outlined in Table 1. Included articles were published in the period 1993–2014. There was a total study population of 4,487,662 individuals, 17,945 of whom had been diagnosed with liver cancer, with 328,420 exposed to gallstones and 884,507 exposed to cholecystectomy. The majority of the studies (*n* = 8) were conducted in European populations [23]–[26], [28]–[31]. Three studies were performed in a North American population [18], [19], [27], and four studies were conducted in an Asian population [17], [20]–[22]. Thirteen studies were population based [17]–[22], [24]–[27], [29]–[31], and two were hospital based [23], [28]. Of the included studies, 11 reported an association between gallstones and the risk of liver cancer [17]–[19], [21]–[24], [26], [27], [29], [30]. Twelve studies reported an association between cholecystectomy and the risk of liver cancer [17]–[21], [24]–[29], [31].

**Table 1:** Characteristics of included studies on the association of gallstones, cholecystectomy and risk of liver cancer.

Study	Year	Country	Studydesign	Setting	Averageage, years	Period of observation	Exposure	Number of Exposure	Total LCcases	All subjectsTotal	Adjustmentfactors	QS
Vogtmannet al. [17]												
SWHS cohort♯	2014	China	Cohort	PB	54.2	2000–2010	Gallstones	8161	160	73209	1–12	7
							Cholecystectomy	3151				
SMHS cohort§	2014	China	Cohort	PB	57.4	2002–2010	Gallstones	4614	252	61337	1–11	7
							Cholecystectomy	1684				
Nogueira, et al. [18]	2014	US	Case control	PB	76.5	1992–2005	Gallstones	15097	10219	1,238,390	7, 13	6
							Cholecystectomy	9109				
Nogueira, et al. [19]	2013	US	Cohort	PB	NR	NR	Gallstones	30,674	414	487,207	1, 2, 4, 5, 9,10, 14–16	2
							Cholecystectomy	25,457				
Kao, et al. [20]	2013	China	Cohort	PB	66.0	1996–2008	Cholecystectomy	2590	67	1,002,590	1, 14	7
Chen, et al. [21]	2013	China	Cohort	PB	55.0	2000–2010	Gallstones	15545	791	77,725	1, 7, 12, 14,17–20	7
							Cholecystectomy	5850				
Chang, et al. [22]	2013	China	Case control	PB	NR	2004–2008	Gallstones	1484	2978	14890	1, 7, 8, 14,18, 19, 20	8
Tavani, et al. [23]	2012	Italy andSwitzerland	Case control	HB	60	1982–2009	Gallstones	206	684	2640	1, 2–5, 14, 21–25	6
Nordenstedt, et al. [24]	2012	Sweden	Cohort	PB	59.9	1965–2008	Gallstones	192,960	170	538211	1, 13, 14	6
							Cholecystectomy	345,251				
Lagergren, et al. [25]	2011	Sweden	Cohort	PB	52.0	1965–2008	cholecystectomy	345,251	333	345,251	1, 13, 14	6
Welzel, et al. [26]	2007	Denmark	Case control	PB	NR	1978–1991	Gallstones	35	764	3820	1, 14, 21	7
							Cholecystectomy	25				
Welzel, et al. [27]	2007	US	Case control	PB	79.0	1993–1999	Gallstones	4445	535	103317	1, 14, 15,26, 27	6
							Cholecystectomy	1690				
Goldacre, et al. [28]	2005	UK	Cohort	HB	15–84	1963–1999	Cholecystectomy	39,254	344	374067	1, 13, 14, 26	6
Chow, et al. [29]	1999	Denmark	Cohort	PB	61.0	1977–1993	Gallstones	17715	82	60176	1, 13, 14	5
							Cholecystectomy	42461				
Johansen, et al. [30]	1996	Denmark	Cohort	PB	60.0	1977–1992	Gallstones	42,098	56	42,098	1, 13, 14	5
Ekbom, et al. [31]	1993	Sweden	Cohort	PB	NR	1965–1987	Cholecystectomy	62,734	96	62,734	1, 13, 14	5

Abbreviations: PB, population based; HB, hospital based; LC, liver cancer; CI, confidence interval; QS: quality score; NR, not report.

♯ Shanghai Women’sHealth Study (SWHS) (1996–2010).

§ Shanghai Men’s Health Study (SMHS) (2002–2010).

Adjustment factors: 1 age, 2 BMI, 3 education, 4 smoking status, 5 alcohol consumption, 6 family history of liver cancer, 7 history of diabetes, 8 history of hepatitis/chronic liver disease, 9 physical activity, 10 total energy intake, 11, income, 12 menopausal status, 13 calendar years, 14 gender, 15 race, 16 non-steroidal anti-inflammatory drugs intake, 17 perlipidemia, 18 hepatitis B virus infection, 19 hepatitis C virus infection, 20 cirrhosis, 21 time of diagnosis, 22, chronic pancreatitis, 23 study center, 24 year of interview, 25 study period, 26 geographic region, 27, state buy-in status.

Table 1 summarizes the methodological quality of all studies, and additional data are given in Table S2. According to the Newcastle-Ottawa scale, most studies were fair (scale of 4–6) to good (scale of 7–9) quality, except the abstract identified from the conference proceedings [19].

### Risk of gallstones in the incidence of liver cancer

**Figure 2:**
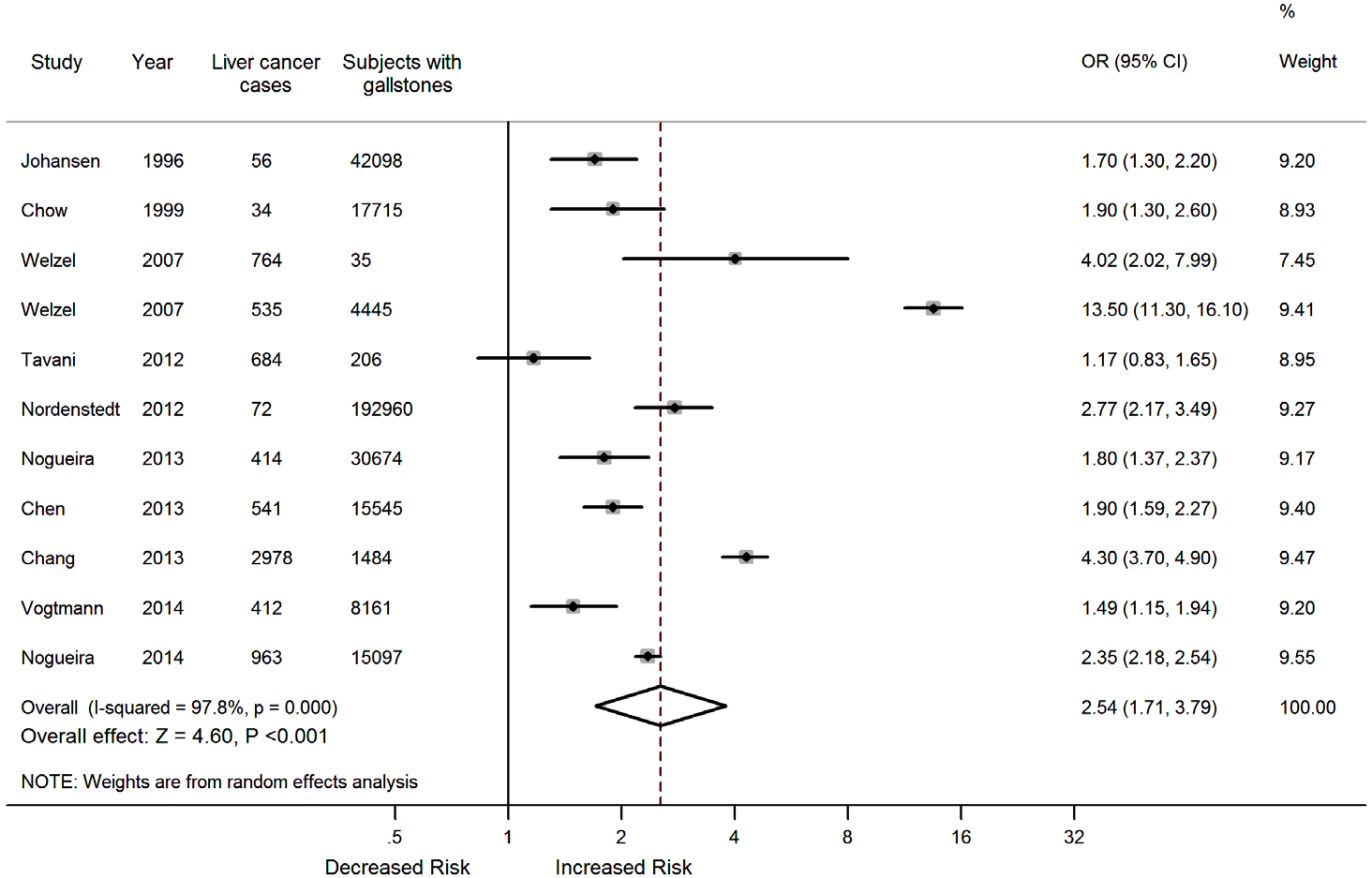
Forest plot of the association between gallstones and risk of liver cancer.

Eleven studies [17]–[19], [21]–[24], [26], [27], [29], [30] reported 7,453 liver cancer events in 328,420 patients with gallstones. The multivariate-adjusted ORs for incident liver cancer with gallstones vs. controls for each study and all studies combined are presented in Fig. 2. The liver cancer incidence was increased in patients with gallstones, with an OR of 2.54 (95% CI, 1.71–3.79) but with considerable heterogeneity (*I^2^* = 97.8%; *P*<0.001).

Some studies [17], [22], [24] provided separate estimates of the OR for liver cancer in male and female gallstone patients. An increased risk of liver cancer in patients with gallstones was seen in females (three studies; adjusted OR, 3.29; 95% CI, 1.02–10.62), as well as in males (three studies; adjusted OR, 2.84; 95% CI, 1.44–5.61), with significant evidence of heterogeneity in both subsets (Table 2).

**Table 2:** Subgroup analysis of odds ratios for the association between gallstones, cholecystectomy and the risk of liver cancer.

Study characteristics	No. ofstudies	Odds ratios (95% CI)	*P_OR_* value	Heterogeneity	
				*I^2^ (%)*	*P* value
**Gallstones**	11	2.54 (1.71, 3.79)	<0.001	97.8	<0.001
** Study Design**					
Case-control studies	5	3.66 (1.75, 7.64)	0.001	98.8	<0.001
Cohort studies	6	1.90 (1.60, 2.25)	<0.001	63.8	0.017
** Study Location**					
Studies in America	3	3.86 (1.15, 12.91)	0.029	99.4	<0.001
Studies in Europe	5	2.02 (1.42, 2.86)	<0.001	81.9	<0.001
Studies in Asia	3	2.31 (1.19, 4.50)	0.013	97.4	<0.001
** studies quality**					
Good (≥7 scores)	4	2.58 (1.44, 4.62)	0.001	96.1	<0.001
Fair and poor (<7 scores)	7	2.51 (1.37, 4.60)	0.003	98.4	<0.001
** Gender**					
Males	3	2.84 (1.44, 5.61)	0.003	93.8	<0.001
Females	3	3.29 (1.02, 10.62)	0.046	97.9	<0.001
**Cholecystectomy**	12	1.62 (1.29, 2.02)	<0.001	91.0	<0.001
** Study Design**					
Case-control studies	3	2.23 (0.73, 6.79)	0.159	97.0	<0.001
Cohort studies	9	1.47 (1.19, 1.81)	<0.001	85.0	<0.001
** Study Location**					
Studies in America	3	2.14 (0.78, 5.88)	0.140	97.0	<0.001
Studies in Europe	6	1.30 (1.20, 1.41)	<0.001	0.0	0.794
Studies in Asia	3	1.76 (0.86, 3.57)	0.120	94.2	<0.001
** studies quality**					
Good (≥7 scores)	4	1.29 (1.21, 1.37)	0.019	95.4	<0.001
Fair and poor (<7 scores)	8	2.32 (1.15, 4.69)	<0.001	0.0	0.911
** Gender**					
Males	4	1.70 (1.05, 2.75)	0.031	90.4	<0.001
Females	4	1.68 (1.00, 2.82)	0.049	91.9	<0.001

We then conducted further subgroup meta-analysis according to study design, study location, and study quality (Table 2). No substantial differences in the summary ORs were found between case-control and cohort studies, good and fair/poor quality studies, as well as studies conducted in Asia and Western country. All of these analyses were with considerable heterogeneity.

We also conducted a meta-regression analysis to investigate the impact of heterogeneous factors on the OR estimates. The study design, study location, and study quality were chosen as the potential heterogeneous factors. In multivariate meta-regression analysis, none of these factors were significant (*P* = 0.200 for study design; *P* = 0.892 for study location; *P* = 0.905 for study quality). The meta-regression analysis indicated that study design, study location, and study quality might not be major sources contributing to the heterogeneity presented in the overall analyses.

We observed no evidence of overly influential studies in sensitivity analyses based on repeatedly computing the pooled ORs, omitting one study at a time. The pooled ORs ranged from 2.13 (1.68–2.70) when the study by Welzel et al. [27] was excluded, to 2.75 (1.81–4.16) when the study by Tavani et al. [23] was excluded.

**Figure 3:**
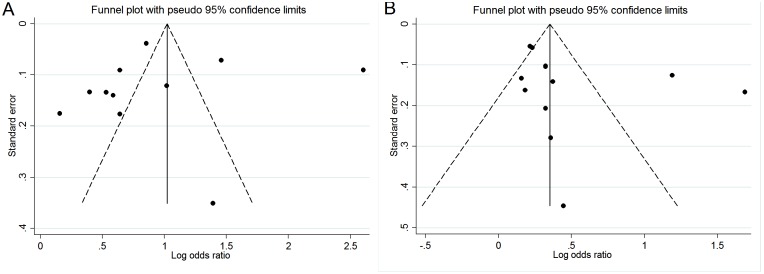
Funnel plot analysis to detect publication bias. A Funnel plot for studies evaluating the association between gallstones and liver cancer risk; B Funnel plot for studies evaluating the association between cholecystectomy and liver cancer risk.

In the publication bias assessment, the inverted funnel plot appeared to be symmetric (Fig. 3A). The *P* value for the Egger test was 0.847, suggesting a very low probability of publication bias.

### Risk of cholecystectomy in the incidence of liver cancer

**Figure 4:**
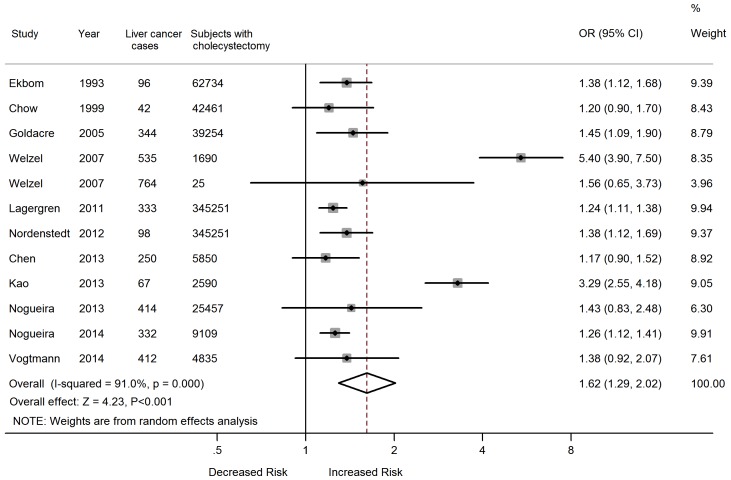
Forest plot of the association between cholecystectomy and risk of liver cancer.

Twelve eligible studies [17]–[21], [24]–[29], [31] were included in the analysis of the potential role of cholecystectomy in the risk of liver cancer. These included 884,507 patients with a history of cholecystectomy and 3,687 liver cancer outcome events. Meta-analysis of these 12 studies showed that compared to individuals without a history of cholecystectomy, those who had their gallbladder removed had a 62% greater risk of liver cancer (OR = 1.62, 95% CI, 1.29–2.02), with considerable heterogeneity among studies; test for heterogeneity (*P*<0.001, *I^2^* = 91.0%) (Fig. 4).

Four studies reported sex-specific risk estimates of liver cancer [17], [20], [24], [25]. No difference was observed in the risk of liver cancer between males (OR = 1.70; 95% CI, 1.05–2.75) and females (OR = 1.68; 95% CI, 1.00–2.82) with a history of cholecystectomy. There was considerable heterogeneity observed in both analyses (Table 2).

When stratifying the data into subgroups based on study design, study location and study quality (Table 2), we found a significant association between cholecystectomy and risk of liver cancer among cohort studies (OR = 1.47) and studies conducted in Europe (OR = 1.30). On restricting analysis to high-quality studies (≥7 scores), we observed a similar association between cholecystectomy and risk of liver cancer (OR = 1.29; 95% CI, 1.21–1.37). However, a non-significant increased risk of liver cancer in patient with cholecystectomy was observed in case-control studies (OR = 2.23; 95% CI, 0.73–6.79), studies conducted in America (OR = 2.14; 95% CI, 0.78–5.88) and in Asia (OR = 1.76; 95% CI, 0.86–3.57).

We then conducted a meta-regression analysis to investigate the impact of study design, study location, and study quality on the estimated ORs. In multivariate meta-regression analysis, study region and study quality were significant factors (*P* = 0.039 and *P* = 0.008, respectively), with these two variables explaining most between-study variability.

In sensitivity analyses, the pooled OR remained significantly increased when studies were excluded one at a time, with the pooled OR ranging from 1.44 (1.22–1.70) when the study by Welzel et al. [26] was excluded, to 1.67 (1.31–2.12) when the study by Chen et al. [21] was excluded.

The shape of the funnel plots for studies on the association between cholecystectomy and liver cancer risk seemed somewhat asymmetrical. However, the *P*-value of Egger’s regression test (*P* = 0.581) was more than 0.05, indicating no statistical evidence of publication bias (Fig. 3B).

## Discussion

To the best of our knowledge, this is the first comprehensive meta-analysis of observational studies to investigate the risk of liver cancer in gallstone patients and cholecystectomy patients. The results of the present meta-analysis of 15 studies point to significant evidence for an increased risk of liver cancer among gallstone patients as compared to those without gallstones (OR = 2.54). In the current study, cholecystectomy was associated with a 62% excess risk of liver cancer. The association persisted across a broad range of sensitivity analyses. Further, the significant association was observed in both women and men. However, there was considerable heterogeneity among most analyses.

There is a long-standing debate about the risk of cancer in patients who have gallstones and undergo cholecystectomy. Several review studies have discussed the potential risk of gallstones or cholecystectomy in various tumors, such as colorectal [42] and colonic adenomas [43], and in several types of cancer, such as colorectal [5], [7], [8], pancreatic [6], esophageal, and gastric [44]. In 1993, Giovannucci et al. found a 34% increased risk of colorectal cancer following cholecystectomy based on combined results from 33 case-control studies [8]. In addition, an analysis by Lin et al. of 18 studies found that cholecystectomy was associated with a 23% excess risk of pancreatic cancer [6]. In contrast, other studies found no effect of cholecystectomy on the risk of colorectal adenoma [42], esophageal and gastric cancer [44], rectal cancer [7] and colonic adenoma [43]. However, Chiong et al. reported a statistically significant risk of rectal cancer (OR = 1.33) [5] and colonic adenoma (OR = 2.26) [43] if gallstones were present. To date, several epidemiological studies have investigated the relationship between gallstones, cholecystectomy, and the risk of liver cancer, but no definitive conclusions have been drawn. The results of our comprehensive meta-analysis of 15 studies, which included 4,487,662 participants and 17,945 liver cancer cases, suggest that gallstones and cholecystectomy might be important contributors to the risk of liver cancer. An understanding of the clinicopathological development of liver cancer is essential for effective screening.

The pathophysiology of tumorigenesis associated with gallstones and after cholecystectomy has yet to be elucidated. One potential mechanism may involve chronic inflammation. Gallstones may induce biliary inflammation, and cholecystectomy is typically followed by dilation of the common bile ducts and elevated bile duct pressure [9], both of which might cause chronic inflammation. The link between chronic inflammation and cancer is well established. In the microenvironment, chronic inflammation can stimulate the release of cytokines, chemokines, growth factors, reactive oxygen species, and reactive nitrogen intermediates, all of which are important constituents of the local environment of tumors [10]. Inflammatory responses also play decisive roles in the cancer development, including initiation, promotion, invasion, and metastasis [45]. Conversely, tumor-related inflammation contributes to further production of reactive oxygen species, reactive nitrogen intermediates, and cytokines [10]. Another hypothesis for the pathophysiology of tumorigenesis associated with gallstones is that removal of the gallbladder results in the accumulation of bile, and secondary bile acids, in particular deoxycholic acid [11]–[13], with the bile acids acting as carcinogens [16]. Gallstones are thought to block the flow of bile through the cystic duct.

Heterogeneity is a potential problem when interpreting the results of all meta-analyses, and finding the sources of heterogeneity is one of the most important goals of any meta-analysis [46]. In our meta-analysis, considerable heterogeneity was observed in most of the analyses. To investigate the sources of heterogeneity, we performed subgroup and meta-regression analyses. In the gallstone studies, despite stratifying the data into subgroups based on study design, study location, study quality, and gender, significant heterogeneity was still detected. The multivariate meta-regression analysis also suggested that none of these variables could explain the heterogeneity observed in the overall analysis. With respect to cholecystectomy, subgroup analyses by study design, study location, and study quality indicated that heterogeneity still existed in the case-control studies, cohort studies, U.S. studies, Asian studies, and high-quality score studies. There was little evidence of heterogeneity in the subgroup analyses of studies in Europe (*P* = 0.794, *I^2^* = 0.0%) and low-quality studies (*P* = 0.911, *I^2^* = 0.0%). To further investigate the heterogeneity, multivariate meta-regression analyses were performed. Meta-regression analysis of the data showed that the study region and study quality might substantially influence the initial heterogeneity. When these two parameters were considered together, the results indicated that the U.S. and Asian studies might be a major source of the heterogeneity in the cholecystectomy data.

The presence of heterogeneity in study design, population characteristics, sample size, information collection methods (e.g., questionnaires or medical records), definition of exposure, assessment of outcome, period of observation, and duration of study follow-up is not surprising. For example, in the 15 studies included in the present meta-analysis, the period of observation was from 1963 to 2010. The older studies [23], [25], [28]–[30] did not seem to be adequately powered to detect a significant difference in liver cancer incidence following gallstones and cholecystectomy, with a low risk ratio reported in these studies. The period of observation of these studies was up to 36 years (from 1963 to 1999). As the most important etiology of primary liver cancer is hepatitis B or C virus infection, we suspect that the low risk ratio may be due to the exclusion of hepatitis C virus infection in the older studies. We suspect that the low risk ratio may be due to without exclusion of hepatitis C virus infection. Thus, pooled effect estimates based on heterogeneous data should be interpreted with caution.

Our study has several strengths. First, our meta-analysis included 15 studies, involving 4,487,662 participants and 17,945 liver cancer cases. The large sample size afforded increased statistical power. We were also able to carry out multiple subgroup analyses and evaluate heterogeneity and the presence of publication bias. Moreover, the present meta-analysis included an approved quality evaluation system. Thus, it minimized potential bias. Further, the likelihood of important selection or publication bias in our meta-analysis is small.

However, several limitations should be acknowledged when interpreting our findings. First, there was significant evidence of considerable heterogeneity among almost all the analyses, despite stratifying the data into subgroups based on several variables. Second, all studies included in our analysis were observational studies, which have inherent limitations. Therefore, confounding factors and bias might have distorted the results [47]. In particular, in the case-control studies, we cannot rule out the possibility of recall bias. Third, as shown in Table 1, the number and content of the adjusted confounders differed between studies. The established risk factors for liver cancer include hepatitis B/C virus infection, contamination of foodstuff with aflatoxins, smoking, alcohol, liver cirrhosis, and diabetes [3], [4]. Most studies [17], [19]–[31] adjusted for age and gender using multivariate statistical models. Some studies [17], [19], [21]–[23] adjusted for smoking, alcohol, liver cirrhosis, diabetes, and family history of liver cancer. Only a few studies [21], [22] adjusted for hepatitis B or C virus infection. The effects of gallstones and cholecystectomy on the risk of liver cancer may be overestimated if other risk factors for liver cancer are imprecisely measured. However, researchers do not always consider the same factors to be potential confounders. It is also almost impossible to obtain and analyze effects estimates extracted from homogeneous models. We believe that selecting the most multivariable-adjusted effects estimates in our meta-analysis minimized the effects of residual confounding. Fourth, publication bias is a problem when interpreting our results. Negative studies are less likely to be published in indexed journals, leading to potential publication bias. We saw no evidence of such publication bias in the Egger’s linear regression test, but the funnel plot seemed asymmetrical. However, according to the *Cochrane Handbook for Systematic Reviews of Interventions*, Egger's teat typically has low power. Thus, even when they do not provide evidence of funnel plot asymmetry, bias, including publication bias, cannot be excluded. Thus, publication bias remains one possible alternative explanation for our positive finding of an association between gallstones and cholecystectomy and an increased risk of liver cancer.

In conclusion, although this meta-analysis has some limitations, particularly the heterogeneity of the studies, the results suggest that a history of gallstones and cholecystectomy is associated with an increased risk of liver cancer. This issue would probably be best addressed using well-designed prospective studies with adequate exposure measurements, accurate case definition, and careful adjustment for major confounders.

## Supporting Information

Table S1 **References for studies excluded from the full text studies review.**
(DOC)

Table S2 **Newcastle–Ottawa scale for assessment of study quality.** A case control studies; B cohort studies.
(DOC)

Checklist S1 **PRISMA check list.**
(DOC)
