# Supplementary material for: Risk of Primary Liver Cancer Associated with Gallstones and Cholecystectomy: A Meta-Analysis
Source: PLoS One. 2014 Oct 7;9(10):e109733. doi: 10.1371/journal.pone.0109733 (PMC4188756; doi:10.1371/journal.pone.0109733)
Supplement: Table S1 — References for studies excluded from the full text studies review. (DOC) [file pone.0109733.s001.doc]

**Table S1. References for studies excluded from the full text studies review**

**Excluded Studies: categorized by reasons for exclusion**

**The outcome of liver cancer not reported (n = 5)**

1. Yen S, Hsieh CC, MacMahon B (1987) Extrahepatic bile duct cancer and smoking, beverage consumption, past medical history, and oral-contraceptive use. Cancer 59: 2112-2116.
2. Kato K, Akai S, Tominaga S, Kato I (1989) A case-control study of biliary tract cancer in Niigata Prefecture, Japan. Jpn J Cancer Res 80: 932-938.
3. Urbach DR, Swanstrom LL, Khajanchee YS, Hansen PD (2001) Incidence of cancer of the pancreas, extrahepatic bile duct and ampulla of Vater in the United States, before and after the introduction of laparoscopic cholecystectomy. Am J Surg 181: 526-528.
4. Welzel TM, McGlynn KA, Hsing AW, O'Brien TR, Pfeiffer RM (2006) Impact of classification of hilar cholangiocarcinomas (Klatskin tumors) on the incidence of intra- and extrahepatic cholangiocarcinoma in the United States. J Natl Cancer Inst 98: 873-875.
5. Hsing AW, Gao YT, McGlynn KA, Niwa S, Zhang M, et al. (2007) Biliary tract cancer and stones in relation to chronic liver conditions: A population-based study in Shanghai, China. Int J Cancer 120: 1981-1985.

**The outcome was cancer mortality (n = 2)**

1. Ichimiya H, Kono S, Ikeda M, Tokudome S, Nakayama F, et al. (1986) Cancer mortality among patients undergoing cholecystectomy for benign biliary diseases. Jpn J Cancer Res 77: 579-583.
2. Shibata A, Ogimoto I, Kurozawa Y, Nose T, Yoshimura T, et al. (2003) Past medical history and risk of death due to hepatocellular carcinoma, univariate analysis of JACC study data. Kurume Med J 50: 109-119.
