# Supplementary material for: Risk of Primary Liver Cancer Associated with Gallstones and Cholecystectomy: A Meta-Analysis
Source: PLoS One. 2014 Oct 7;9(10):e109733. doi: 10.1371/journal.pone.0109733 (PMC4188756; doi:10.1371/journal.pone.0109733)
Supplement: Table S2 — Newcastle–Ottawa scale for assessment of study quality. A case control studies; B cohort studies. (DOC) [file pone.0109733.s002.doc]

**Table S2 A** Newcastle–Ottawa scale for assessment of quality of case control studies (each asterisk represents if individual criterion within the subsection was fulfilled)

| **NOS scale** | Acceptable(★) |  |  |  |  |  |  |
| --- | --- | --- | --- | --- | --- | --- | --- |
| **A Selection (maximum 4)** |  | Nogueira, 2014 | Nogueira, 2013 | Chang, 2013 | Tavani, 2012 | Welzel,2007 | Welzel,2007 |
| 1 Case definition adequate | With independent validation | ★ | – | ★ | ★ | ★ | – |
| 2 Representativeness of the cases | Consecutive or obviously representative series of cases | ★ | – | ★ | ★ | ★ | ★ |
| 3 Selection of controls | Community controls | ★ | – | ★ | – | ★ | ★ |
| 4 Definition of control | No history of liver cancer | ★ | – | ★ | ★ | ★ | ★ |
| **B Comparability (maximum 2)** |  |  |  |  |  |  |  |
| 1 Comparability of cases and controls on the basis of the design or analysis | Study controls for age and sex | – | ★ | ★ | ★ | ★ | ★ |
| 2 Study controls for at least 3 additional risk factors | Hepatitis B/C virus infection, smoking, alcohol, family history of liver cancer, α-fetoprotei level, diabetes, body mass index, age, gender | – | ★ | ★ | ★ | – | – |
| **C Exposure (maximum 3)** |  |  |  |  |  |  |  |
| 1 Ascertainment of exposure | Secure record or structured interview where blind to case/control status | ★ | – | ★ | – | ★ | ★ |
| 2 Same method cases and controls | Yes | ★ | – | ★ | ★ | ★ | ★ |
| 3 Non-response rate | Same rate for both groups | – | – | – | – | – | – |
| **Totaal (maximum 9)** |  | **6** | **2** | **8** | **6** | **7** | **6** |

**Table S2B** Newcastle–Ottawa scale for assessment of quality of cohort studies (each asterisk represents if individual criterion within the subsection was fulfilled)

| Quality assessment criteria | Acceptable(★) |  |  |  |  |  |  |  |  |  |
| --- | --- | --- | --- | --- | --- | --- | --- | --- | --- | --- |
| **Selection** |  | Vogtmann, 2014 | Kao, 2013 | Chen, 2013 | Nordenstedt, 2012 | Lagergren, 2011 | Goldacre, 2005 | Chow, 1999 | Johansen, 1996 | Ekbom, 1993 |
| Representativeness of the exposed cohort? | Representative of average adult in community (age/sex/being at risk of disease) | ★ | ★ | ★ | ★ | ★ | ★ | ★ | ★ | ★ |
| Selection of the non exposed cohort? | Drawn from same community as exposed cohort | ★ | ★ | ★ | – | – | ★ | – | – | – |
| Ascertainment of exposure | Secured records, structured interview | – | ★ | ★ | ★ | ★ | ★ | ★ | ★ | ★ |
| Demonstration that outcome of interest was not present at start of study | Only incident cases of liver cancer | ★ | ★ | – | ★ | ★ | – | – | – | – |
| **Comparability** |  |  |  |  |  |  |  |  |  |  |
| Comparability of cohorts on the basis of the design or analysis | Study controls for age and sex | ★ | ★ | ★ | ★ | ★ | ★ | ★ | ★ | ★ |
| Study controls for at least 3 additional risk factors? | Hepatitis B/C virus infection, smoking, alcohol, liver cirrhosis, obesity, diabetes, body mass index, age, gender | ★ | – | ★ | – | – | – | – | – | – |
| **Outcome** |  |  |  |  |  |  |  |  |  |  |
| Assessment of outcome | Independent blind assessment, record linkage | ★ | ★ | ★ | ★ | ★ | ★ | ★ | ★ | ★ |
| Was follow-up long enough for outcomes to occur | Follow-up ≥4 years | ★ | ★ | ★ | ★ | ★ | ★ | ★ | ★ | ★ |
| Adequacy of follow up of cohorts | Complete follow-up, or subjects lost to follow-up unlikely to introduce bias (>90% follow up, or description provided of those lost) | – | – | – | – | – | – | – | – | – |
| **Overall quality score (maximum = 9)** |  | **7** | **7** | **7** | **6** | **6** | **6** | **5** | **5** | **5** |
